# An MYB-Related Transcription Factor, UpMYB-PHL, Is Involved in Salt Tolerance by Coordinating Phosphorus Transporter and Energy Metabolism in *Ulva prolifera*

**DOI:** 10.3390/biology15131050

**Published:** 2026-07-01

**Authors:** Xiuwen Yang, Jiahui Xu, Hongyan He, Songdong Shen

**Affiliations:** School of Basic Medical Sciences, Soochow University, Suzhou 215101, China; xwyang@suda.edu.cn (X.Y.); jhxu98@stu.suda.edu.cn (J.X.); hyhe5027@suda.edu.cn (H.H.)

**Keywords:** *Ulva prolifera*, salt stress, MYB-related transcription factor, phosphate transporter, energy metabolism

## Abstract

*Ulva prolifera* is the main causative species of marine green tides and exhibits extreme tolerance to intertidal abiotic stress. However, the underlying molecular mechanisms remain largely unclear. In this study, we cloned and characterized an MYB-related transcription factor, UpMYB-PHL, from *U. prolifera*. Expression analysis showed that *UpMYB-PHL* is rapidly and significantly induced by high-salt stress. Furthermore, heterologous overexpression of *UpMYB-PHL* in the model microalga *Chlamydomonas reinhardtii* significantly improved its salt tolerance and biomass. By yeast one-hybrid and dual-luciferase assays, we demonstrated that UpMYB-PHL directly binds to and activates the promoter of the phosphate transporter gene *UpPHT1*, which is a typical target gene of phosphate response (PHR) transcription factor and participates in salt stress responses of plants. Interestingly, yeast two-hybrid assays revealed that UpMYB-PHL physically interacts with UpGAPDH, a core enzyme in energy metabolism. Taken together, our findings reveal a novel regulatory network in which UpMYB-PHL coordinates phosphorus transporter and energy metabolism in response to salt stress in *U. prolifera*. This study provides a vital molecular explanation for the rapid adaptation and massive growth of *U. prolifera* under severe intertidal salt stress.

## Simple Summary

Since 2007, the periodic excessive proliferation of *Ulva prolifera* in the Yellow Sea of China has had a significant impact on the ecological environment. Their astonishing abiotic stress tolerance is regarded as one of the reasons why they can thrive in harsh environments. In this study, we characterized an MYB-related transcription factor from *U. prolifera*, namely UpMYB-PHL, which has been proven to be involved in the salt stress response of algae. We also reveal how UpMYB-PHL-coordinates phosphorus transporter and energy metabolism in response to salt stress in *U. prolifera.* The results of this study provide a new perspective for understanding the salt stress tolerance of *U. prolifera*.

## 1. Introduction

Green tide is an abnormal marine ecological phenomenon caused by floating green macroalgae’s explosive proliferation and rapid accumulation under appropriate environmental conditions [1]. Since 2007, the green tide has occurred periodically in the Yellow Sea of China, causing serious damage to the coastal marine ecosystem, aquaculture and tourism [2,3]. *Ulva prolifera*, the dominant species of green tide, is attributable not only to its rapid proliferation, but also to its remarkable tolerance to abiotic stress [4,5,6]. *U. prolifera* usually inhabit the intertidal zone, and are subjected to dramatic environmental changes during the growth and development process [7,8]. In particular, during the low tide and green tide outbreak periods, the thalli of *U. prolifera* are exposed to air for several hours, which is always accompanied by severe comprehensive stress, such as drought stress and salt stress [9]. Among these, salt stress is one of the crucial limiting factors for its growth [10]. Currently, it has been reported that salinity and temperature play important roles in driving *U. prolifera* outbreaks [11].

To date, there is little understanding of *U. prolifera*’s response to salt stress. For example, when exposed to salt stress, *U. prolifera* rapidly generates hydrogen peroxide (H_2_O_2_), one of the reactive oxide species (ROS), thereby inducing oxidative stress [12]. However, *U. prolifera* has evolved multiple defense systems for oxidative stress, through enzymes, such as catalases (CATs), superoxide dismutases (SODs) and peroxidases (PRXs), as well as non-enzymatic, i.e., glucose, fucoidan and zeaxanthin [13]. For instance, zeaxanthin epoxidase (ZEP) plays a significant role in *U. prolifera*’s response to high-salt stress [14]. And UpSnRK2 directly enhances the MnSOD activity by coordinating the PP2C-SnRK2-MnSOD module, thereby regulating the ROS balance and increasing *U. prolifera*’s tolerance to salt stress [15]. Although these studies have provided certain biological insights into *U. prolifera*’s response to salt stress, the mechanism underlying this tolerance still requires further investigation.

Interestingly, during our previous study on the salt tolerance of *U. prolifera*, we discovered an MYB-related protein that responded actively to salt stress. We speculate that it might play a role in salt stress tolerance in *U. prolifera*. MYB-related proteins are members of the MYB transcription factors (TFs) superfamily, which is characterized by the presence of intact or partial MYB repeats (R) in the MYB DNA-binding domain [16]. And the MYB-related family comprises heterogeneous proteins with fewer conserved motifs outside the MYB domain, which can be classified into several subfamilies [16,17]. Massive MYB-related family genes have been discovered in diverse plants, and they play a key role in development processes and the response to abiotic stresses [18,19]. With evolution, the number of MYB-related genes has increased in plants, but they remain highly conserved from unicellular algae to vascular plants [20]. In higher plants, such as *Arabidopsis thaliana* and rice, the MYB-related TF is a central regulator of abiotic stress response, such as Pi starvation, and salt and drought stress [16,20]. In green algae *Chlamydomonas reinhardtii*, MYB-related TFs PSR1 regulates the expression of numerous genes involved in the response to phosphate (Pi) starvation [21]. However, the MYB-related proteins in other algae still need to be explored. For example, in red algae *Pyropia yezoensis*, 13 MYB-related members were identified, and they were believed to be involved in stress-responsive processes [22]. In *U. prolifera*, an R2R3-MYB TF is regarded as modulating the expression of carotenoid biosynthesis genes [23]. However, the functions and regulatory mechanisms of MYB-related TFs have not yet been revealed in *U. prolifera*.

In this study, the MYB homologous protein designated UpMYB-PHL was isolated from *U. prolifera*; it is essentially a transcription factor. And its role and regulatory mechanism were analyzed. Firstly, the function of UpMYB-PHL was detected through RT-qPCR and heterologous overexpression analysis. Next, the downstream target genes and potential interacting proteins of UpMYB-PHL were screened at genome-wide level. Combined with in vitro and in vivo experiments, the cis-regulatory elements and transcriptional activation of the downstream target genes was anatomized, so as to identify the candidate interacting proteins. This study aims to provide theoretical and data-driven support for a deeper understanding of *U. prolifera*’s response to salt stress and offer guidance for the green tide’s prevention.

## 2. Materials and Methods

### 2.1. Materials, Growth Conditions and Treatments

*Ulva prolifera* was collected from Qingdao, China (36°48′39.75″ N, 121°38′10.88″ E), and subsequently cultured in the laboratory. In the laboratory, *U. prolifera* was cultured in sterile seawater-equipped von Stoch’s enriched (VSE) medium at 20 °C under a light intensity of 100 μmol photons m^−2^·s^−1^ and a 14:10 h light:dark (L:D) photoperiod [24]. Then, the thalli were subjected to culture in different salinity seawater (the 30‰ control group and 50‰ high-salt group) for 1, 2 and 3 days.

The cell wall-deficient strain *Chlamydomonas reinhardtii* (CC-503) was generously provided by Professor Yingjuan Wang (College of Life Science, Northwest University, Xi’an, China). *C. reinhardtii* was cultured in Tris-acetate-phosphate (TAP) medium [14]. The culture conditions were set at 25 °C under a light intensity of 50 μmol photons m^−2^·s^−1^ and a 10:14 h light:dark photoperiod with shaking at 100 rpm. After pre-culture treatment, the strains were cultured in a different conditional medium (the normal TAP medium or containing 120 mmol·L^−1^ sodium chloride (NaCl)) for 0, 1, 2, 3, 4 and 5 days. The growth rate was determined by measuring the optical density at 750 nm (OD_750_) using UV-VIS spectrophotometer (METASH UV-6000) (Metash, Shanghai, China).

### 2.2. Cloning and Bioinformatic Analysis of UpMYB-PHL

The predicted sequence of the *UpMYB-PHL* gene was retrieved from the *U. prolifera* genome database (https://www.ncbi.nlm.nih.gov/datasets/genome/GCA_004138255.1/, accessed on 6 June 2026) [25]. According to the sequence, primers for cloning the *UpMYB-PHL* gene were designed by Primer Premier 3. The primer sequences are provided in Supplementary Table S1. The full-length sequence and coding DNA sequence (CDS) of the *UpMYB-PHL* gene were obtained by PCR reaction using genomic DNA and cDNA of *U. prolifera* as templates, respectively. Then, the conserved domain of UpMYB-PHL was analyzed using the CD-Search tool (https://www.ncbi.nlm.nih.gov/Structure/cdd/wrpsb.cgi/, accessed on 6 June 2026) on the National Center for Biotechnology Information (NCBI). Multiple sequence alignment of MYB DNA-binding and MYB CC domain across species was performed using ClustalW2.1. To elucidate the evolutionary relationship of UpMYB-PHL, highly homologous protein sequences from representative higher plants and the green algae were downloaded from the NCBI database. Moreover, a phylogenetic tree was constructed using the neighbor-joining (NJ) method in MEGA11. Then, the Newick tree was visualized and annotated using the iTOL online tool (https://itol.embl.de/, accessed on 6 June 2026).

### 2.3. RNA Isolation and qRT-PCR Assays

Total RNA isolation and qRT-PCR assays were performed as previously described [14]. Briefly, total RNA was isolated from *U. prolifera* samples using a plant RNA extraction kit (Vazyme Biotechnology, Nanjing, China). Reverse transcription was performed with a Hifair^®^ II 1st Strand cDNA Synthesis Kit (Yeasen Biotechnology, Shanghai, China). Then, qRT-PCR was performed using Hieff^®^ qPCR SYBR Green Master Mix (Low Rox Plus) (Yeasen Biotechnology, Shanghai, China). The qRT-PCR primer sequences are listed in Supplementary Table S1, and 18S rRNA gene was used as the internal reference. Three biological replicates were used for each sample, and the qRT-PCR data were analyzed using the 2^−ΔΔCT^ method.

### 2.4. Plasmid Construction and C. reinhardtii Transformation Assays

The pChlamy3 vector was kindly provided by Professor Wenlei Wang (Fisheries College, Jimei University, Xiamen, China). The *UpMYB-PHL-His* fragment was inserted into the pChlamy3 vector to construct the recombinant vector pChlamy3-*UpMYB-PHL-His*. The recombinant vector was introduced into the *C. reinhardtii* via square-wave electroporation [26]. Selection was carried out by transferring the transformants onto TAP solid medium supplemented with 20 μg/mL hygromycin B (Sangon, Shanghai, China). Positive transformants were grown in 10 mL TAP medium, followed by protein extraction. Western blot analysis was carried out with anti-His antibody (Beyotime, Shanghai, China). The primers used for the assays are listed in Supplementary Table S1.

### 2.5. Evaluation of the Salt Tolerance of UpMYB-PHL-Overexpression Transgenic C. reinhardtii

The wild-type (WT) and *UpMYB-PHL*-overexpression (*UpMYB-PHL*-OE) transgenic *C. reinhardtii* were inoculated into liquid TAP medium supplemented with 120 mmol·L^−1^ NaCl. All cell lines were adjusted to an initial OD_750_ of 0.3. To evaluate the salt tolerance of the transgenic *C. reinhardtii*, cell growth was monitored daily by measuring OD_750_.

To investigate the expression pattern of *UpMYB-PHL* under continuous salt stress, the transgenic *C. reinhardtii* were cultured in TAP medium containing 120 mmol·L^−1^ NaCl at an initial OD_750_ of 0.3. To ensure consistent cell numbers across different growth stages, a volume of algal culture calculated as 5/OD_750_ mL was harvested following each dailyOD_750_ measurement. Total proteins were then extracted from the collected pellets and analyzed by Western blotting to monitor the dynamic changes in UpMYB-PHL protein abundance.

### 2.6. Screening and Verification for UpMYB-PHL Interacting Proteins by Yeast Two-Hybrid (Y2H) Assay

To identify the interacting proteins, UpMYB-PHL was used as a bait to screen from the *U. prolifera* cDNA library though Y2H assay. First, the pGBKT7-*UpMYB-PHL* and pGADT7 vectors were co-transformed into *Saccharomyces cerevisiae* strain AH109 to assess the autoactivation of UpMYB-PHL. Then, the pGADT7-cDNA vector was introduced into yeast AH109 harboring pGBKT7-*UpMYB-PHL* and subsequently cultured on SD/-Trp/-Leu and SD/-Trp/-Leu/-His/-Ade plates (Coolaber, Beijing, China) to screen for UpMYB-PHL-interacting proteins. Positive single clones were verified by sequencing. Finally, the pGBKT7-*UpMYB-PHL* and pGADT7-*UpGAPDH* vectors were co-transferred into yeast AH109, and the transformants selected on an SD/-Leu/-Trp plate (Coolaber, Beijing, China). Positive single clones were picked and serially diluted with water and spotted onto SD/-Trp/-Leu and SD/-Trp/-Leu/-His/-Ade plates (Coolaber, Beijing, China). The plates were incubated at 30 °C for 3–5 days, after which the transformants’ growth was recorded. The pGBKT7-T and pGADT7-lam vectors were co-transformed into yeast AH109 as a negative control. The pGBKT7-T and pGADT7-p53 vectors were co-transformed into yeast AH109 (Coolaber, Beijing, China) as a positive control.

### 2.7. Bioinformatic Analysis and Molecular Cloning of the UpPHT1 Promoter Region from U. prolifera

The upstream regulatory region (2000 bp upstream of the start codon) of the *UpPHT1* gene was obtained from the *U. prolifera* genome database. To identify putative UpMYB-PHL binding sites, the promoter sequence of the *UpPHT1* gene was analyzed on the PlantCARE online database (http://bioinformatics.psb.ugent.be/webtools/plantcare/html/, accessed on 6 June 2026) to detect classical cis-acting elements, specifically the P1BS motif. Subsequently, the promoter fragments of the *UpPHT1* gene, *pro1PHT1* (harboring the P1BS motif), *pro2PHT1* (harboring the MYB recognition site) and *pro3PHT1* (harboring the drought-inducible MYB recognition site), were amplified from the genomic DNA.

### 2.8. Yeast One-Hybrid (Y1H) and Dual-Luciferase Reporter (LUC) Assays

To identify whether UpMYB-PHL binds to the *UpPHT1* promoter in vivo, the Y1H assay was conducted. In brief, the *UpPHT1* promoter fragments were cloned into the pAbAi vector to generate the bait construct pAbAi-*proUpPHT1*. Meanwhile, we cloned the full-length CDS of *UpMYB-PHL* into the pGADT7 vector to generate the prey construct pGADT7-*UpMYB-PHL*. The pAbAi-*proUpPHT1* vector was subsequently linearized and transformed into yeast Y1HGold. To assess autoactivation, the transformed bait strain was cultured on SD/-Ura plate supplemented with increasing concentrations of Aureobasidin A (AbA) (Yeasen, Shanghai, China) (0–1000 ng/mL) to determine the optimal inhibitory concentration. Subsequently, the positive strains harboring the bait construct were prepared as competent cells, and the pGADT7-*UpMYB-PHL* vector was transformed into these cells. Finally, the co-transformed yeast cells harboring both vectors were cultured on an SD/-Leu plate (Coolaber, Beijing, China) with the optimal concentration of AbA to verify the protein–DNA interaction.

A dual-luciferase reporter assay was performed to quantify the transactivation ability of UpMYB-PHL on the *UpPHT1* promoter. In brief, the full-length CDS of *UpMYB-PHL* was inserted into the pGreenII 62-SK vector to serve as the effector, while the fragment of the *UpPHT1* promoter was inserted into the pGreenII 0800-Luc vector to serve as the reporter. Subsequently, the pGreenII 62-SK-*UpMYB-PHL* and pGreenII 0800-Luc-*proUpPHT1* vectors were co-transformed into *C. reinhardtii* via square-wave electroporation. The empty pGreenII 62-SK and pGreenII 0800-Luc-*proUpPHT1* vectors were co-transformed into *C. reinhardtii* as a negative control. Finally, the Luc to Ren enzyme activity ratios for all samples were quantified using the Dual-Luciferase Reporter Gene Assay Kit (Yeasen Biotechnology, Shanghai, China) and a BioTek Synergy H1 microplate reader (BioTek, Winooski, VT, USA).

### 2.9. Statistical Analysis

Data were presented as mean ± standard deviation (SD). Statistical analyses were conducted with GraphPad Prism 10 software. The significant differences were analyzed by Student’s *t*-test (* *p* < 0.05, ** *p* < 0.01, *** *p* < 0.001). Three biological replicates were performed for all experimental treatments.

## 3. Results

### 3.1. UpMYB-PHL Encodes a Member of MYB-Related TFs Family

**Figure 1:**
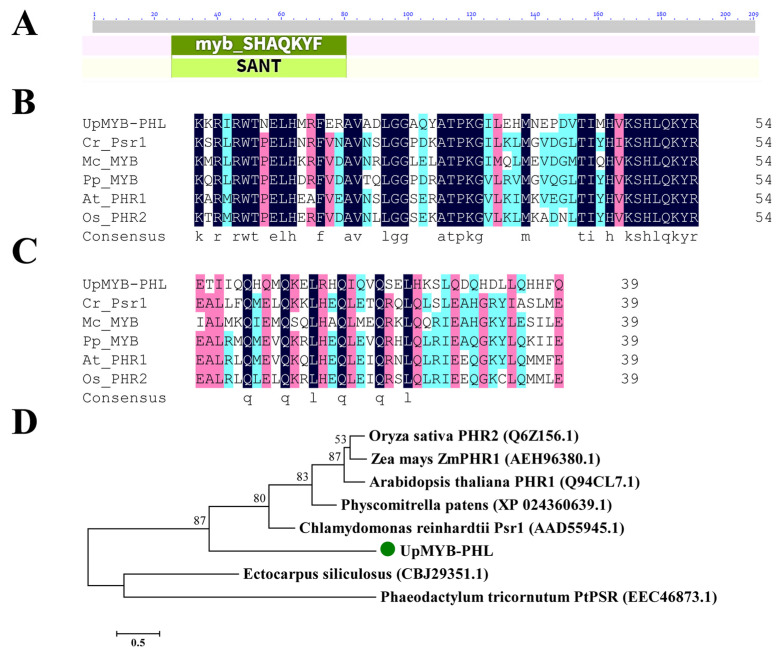
Sequence features and evolutionary conservation of UpMYB-PHL. (**A**) Schematic diagram of the UpMYB-PHL protein structure. The highly conserved MYB DNA-binding domain is highlighted. (**B**,**C**) Multiple sequence alignment of the MYB DNA-binding domain and MYB CC domain from UpMYB-PHL and its orthologs in other species. Identical amino acid residues are highlighted with black background and conserved residues are in pink background. Accession numbers of MYB-related sequences are: AAD55945.1 (Cr Psr1), XP_002504000.1 (Mc MYB), XP_024360639.1 (Pp MYB), Q94CL7.1 (AtPHR1) and Q6Z156.1 (OsPHR2). Cr, *Chlamydomonas reinhardtii*; Mc, *Micromonas commode*; Pp, *Physcomitrella patens*; At, *Arabidopsis thaliana*; Os, *Oryza sativa*. (**D**) Unrooted maximum likelihood (ML) tress based on a protein alignment of *UpMYB-PHL* and homologous MYB-related proteins from selected algae and plants. Numbers at nodes represent bootstrap values based on 1000 replicates. The target protein, UpMYB-PHL from *U. prolifera*, is marked with a green dot. The scale bar represents 0.5 amino acid substitutions per site. The protein sequences for (**D**) are summarized in Table S2.

The MYB TFs are known components of a central regulatory system controlling transcriptional responses to abiotic stress in plants. Previously, we identified and cloned an MYB-related gene *UpMYB-PHL* when exploring the salt stress response of *U. prolifera*. The *UpMYB-PHL* gene has a full-length sequence of 950 bp, consisting of two exons and one intron, and its CDS is 630 bp in length, encoding 209 amino acids. Protein domain prediction revealed that UpMYB-PHL contains a single highly conserved MYB DNA-binding domain with a SHAQKYF motif at 26 to 80 aa (Figure 1A). A putative nuclear localization signal (NLS, KKRIR, amino acid residues 25–30) is present upstream of the MYB DNA-binding domain, which was predicted by LOCALIZER 1.0 [27]. Multiple sequence alignment revealed that UpMYB-PHL shares remarkably high sequence identity with its orthologs, not only in green algae but also in distant land plants (Figure 1B,C). Notably, the key amino acid residues responsible for DNA binding are strictly conserved across these divergent lineages, suggesting a conserved structural basis for targeting downstream genes.

To further elucidate the evolutionary relationships of UpMYB-PHL, a phylogenetic tree was constructed based on representative MYB-related TFs (Figure 1D). The phylogenetic analysis showed that these MYB TFs are distinctly divided into two major clades: the land plant and green algae lineage; moreover, diatom and brown algae lineage. As expected, UpMYB-PHL clustered tightly with orthologs from the land plant and green algae and exhibiting the closest evolution relationship with its homolog from *C. reinhardtii*. Together, UpMYB-PHL is a highly conserved MYB-related TF, suggesting it may retain fundamental regulatory roles in the evolution of photosynthetic eukaryotes.

### 3.2. Heterologous Overexpression of UpMYB-PHL Significantly Enhances Salt Stress Tolerance in C. reinhardtii

**Figure 2:**
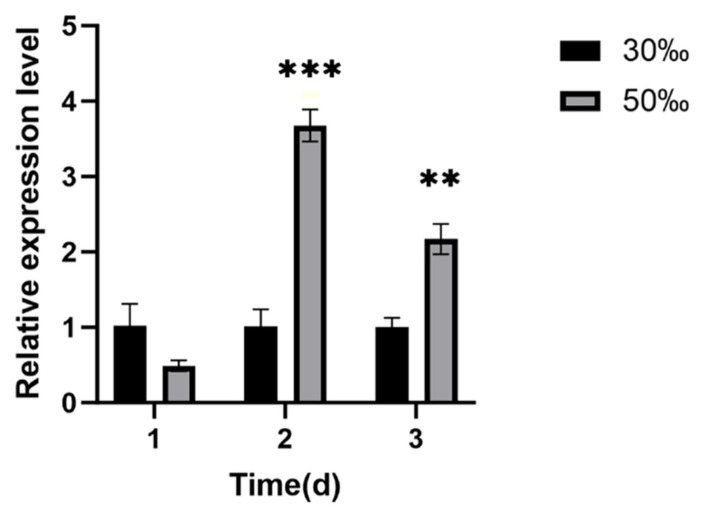
Expression levels of the *UpMYB-PHL* gene in *U. prolifera*. The relative transcript levels of *UpMYB-PHL* were quantified by qRT-PCR. Thalli were cultured under normal salinity (30‰, black bars) and high salinity (50‰, grey bars) conditions for 1, 2 and 3 days. All data are presented as the mean ± standard deviation (SD) of three independent biological replicates (*n* = 3). Asterisks indicate significant differences between the salt-treated group and the control group at each time point (Student’s *t*-test, ** *p* < 0.01, *** *p* < 0.001).

To clarify the role of *UpMYB-PHL* in the response of *U. prolifera* to high-salt stress, we evaluated its expression pattern cultured under high salinity (50‰) and normal salinity (30‰) for 1, 2 and 3 days. As shown in Figure 2, high-salt treatment significantly induced *UpMYB-PHL* transcription. Relative expression peaked at day 2, exhibiting a highly significant upregulation (*p* < 0.001). Although the expression level decreased by day 3, it remained significantly elevated compared with the control (*p* < 0.01).

**Figure 3:**
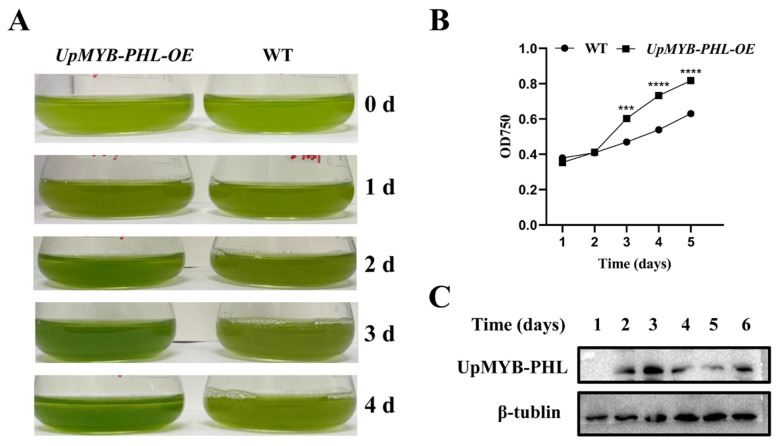
Overexpression of *UpMYB-PHL* enhances salt tolerance in *C. reinhardtii*. WT and *UpMYB-PHL*-OE transgenic *C. reinhardtii* phenotypes (**A**) and growth curves (**B**) in TAP medium supplemented with 120 mmoL·L^−1^ NaCl. Growth was monitored daily by OD_750_ measurements. Data are presented as the mean ± SD (*n* = 3). Asterisks indicate highly significant differences between the WT and transgenic strains at the corresponding time points (Student’s *t*-test, *** *p* < 0.001, **** *p* < 0.0001). (**C**) *UpMYB-PHL* protein levels in transgenic *C. reinhardtii* under continuous salt stress (days 0–5). β-tubulin was used as the internal control protein. The original Western blot images are summarized in File S1.

To further confirm the role of *UpMYB-PHL* in salt stress resistance, we generated *UpMYB-PHL*-overexpression (*UpMYB-PHL*-OE) transgenic *C. reinhardtii*. And the wild-type (WT) and *UpMYB-PHL*-OE *C. reinhardtii* were treated with 120 mmol·L^−1^ NaCl. On days 1 and 2 of the treatment, the color of both the WT and transgenic strains changed from light green to dark green, with no obvious difference between them. However, the transgenic strain became greener than the WT on days 3 and 4 (Figure 3A). Additionally, growth curve analysis revealed that the WT and transgenic strains grew slowly during the first two days of salt stress; however, the transgenic strain exhibited significantly accelerated growth from day 3 (*p* < 0.0001) (Figure 3B). By day 5, the OD_750_ of the transgenic strain had exceeded 0.8, whereas that of the WT was approximately 0.6. The phenotypic observations and biomass measurements were consistent, indicating that overexpression of *UpMYB-PHL* in *C. reinhardtii* effectively alleviates growth inhibition and significantly enhances salt tolerance. The protein abundance of *UpMYB-PHL* under continuous salt stress was monitored by Western blotting to elucidate its molecular response (Figure 3C). Prior to stress (day 0), the internal control *β*-tubulin was clearly detected under normal culture conditions, whereas the UpMYB-PHL band was barely visible, indicating an extremely low basal expression level. Upon salt stress, the protein level of *UpMYB-PHL* exhibited a highly dynamic accumulation pattern. The *UpMYB-PHL* protein was rapidly induced by day 1 and appeared to reach its peak on day 2. Subsequently, the protein level gradually decreased on days 3 and 4, followed by a distinct rebound on day 5. This dynamic profile consistent with the transcript expression pattern and phenotypic adaptation, suggests that *UpMYB-PHL* mediates both the rapid early response and sustained long-term adaptation to prolonged salinity.

### 3.3. UpPHT1 Is Identified as a Candidate Target Gene of UpMYB-PHL

**Figure 4:**
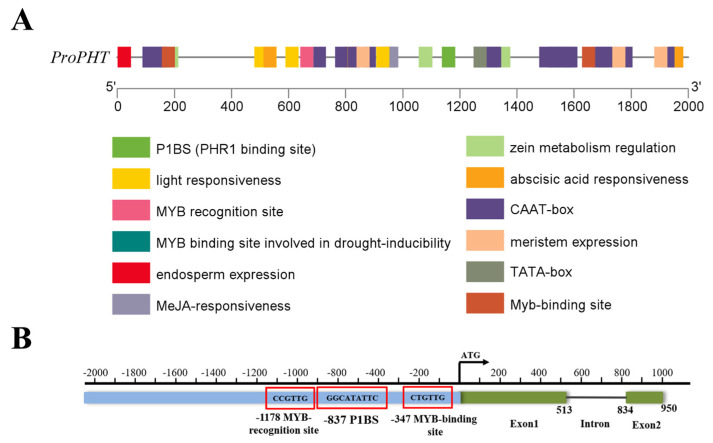
Schematic structure of the *UpPHT1* promoter regions. (**A**) Motifs of the *UpPHT1* promoter. The schematic diagram illustrates the distribution of distinct motifs in the 2000 bp upstream regulatory region of the *UpPHT1* gene (denoted as *ProPHT*). Distinct regulatory motifs identified via the PlantCARE are represented by different colored boxes. (**B**) Location of selected motifs in *UpPHT1* promoter. The motifs are marked in red frames, and the numbers of the scale plates indicate the location from the adenine–thymine–guanine (ATG) start codon.

In plants, MYB-related TFs typically regulate the expression of phosphate transporter 1 (PHT1) family genes to maintain Pi homeostasis. To elucidate the transcriptional regulatory mechanism of UpMYB-PHL, a PHT1 family gene, *UpPHT1*, was isolated from *U. prolifera.* The core element of *UpPHT1* gene promoter was analyzed on the PlantCARE database (Figure 4A). Notably, a classical P1BS cis-acting element (5′-GNATATNC-3′) was identified within the *UpPHT1* promoter region by in silico sequence analysis (Figure 4B). Additionally, a general MYB recognition site motif (5′-CCGTTG-3′) and a drought-inducible MYB-binding site (5′-CAACAG-3′) motif were identified. These results raise the possibility that UpMYB-PHL regulate the *UpPHT1* gene, motivating further investigation to confirm their direct interaction.

### 3.4. UpMYB-PHL Regulates the Expression of UpPHT1 by the P1BS Motif

**Figure 5:**
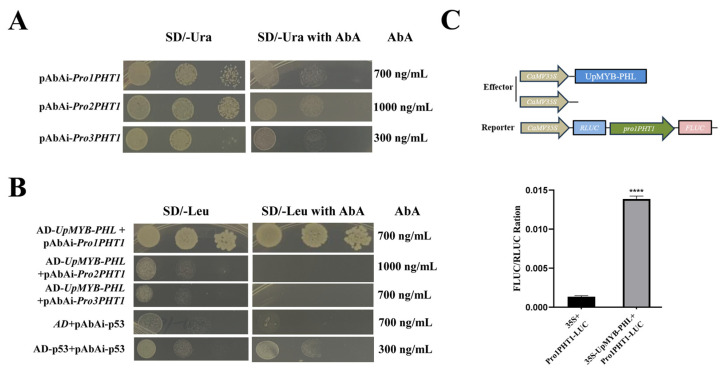
UpMYB-PHL directly binds to the *UpPHT1* promoter through the P1BS motif. (**A**) Assessment of the autoactivation of the *UpPHT1* promoter fragments (*Pro1PHT1*, *Pro2PHT1* and *Pro3PHT1*) in the Y1H system. Basal expression of the reporter genes was assessed on SD/-Ura medium supplemented with gradient concentrations of AbA. The minimal inhibitory concentrations were determined as 700, 1000 and 300 ng/mL for *Pro1PHT1*, *Pro2PHT1* and *Pro3PHT1*, respectively. (**B**) A Y1H assay confirming the specific binding of UpMYB-PHL to the *Pro1PHT1* fragment. The co-transformants harboring the bait and prey vectors were spotted onto SD/-Leu and SD/-Leu/AbA plates. A 10-fold serial dilution of the yeast cells was applied from left to right, starting from an initial OD_600_ of 0.2. (**C**) Dual-luciferase reporter assay. The *Pro1PHT1* fragment, which drives the fLUC reporter, was co-expressed with the UpMYB-PHL effector under the control of the 35S promoter. Renilla luciferase (rLUC) was employed as an internal control. Data are presented as the mean ± SD (*n* = 3). Asterisks indicate highly significant differences (Student’s *t*-test, **** *p* < 0.0001).

To experimentally verify the predicted protein–DNA interaction of UpMYB-PHL and *UpPHT1*, a Y1H assay was conducted using three specific promoter fragments: *pro1PHT1* (harboring the P1BS motif), *pro2PHT1* (MYB-binding site) and *pro3PHT1* (MYB recognition site). To eliminate false positives arising from basal expression, the bait strains (pAbAi-*pro1PHT1*, pAbAi-*pro2PHT* and pAbAi-*pro3PHT1*) were screened on an SD/-Ura plate supplemented with a gradient of AbA concentrations. The optimal concentrations of AbA for inhibiting the *pro1PHT1*, *pro2PHT1* and *pro3PHT1* bait strains were determined to be 700, 1000 and 300 ng/mL, respectively (Figure 5A). Subsequently, the prey construct pGADT7-*UpMYB-PHL* was transformed into these bait strains. As shown in Figure 5B, only the co-transformants harboring the pAbAi-*pro1PHT1* bait and the pGADT7-*UpMYB-PHL* prey showed robust growth on the SD/-Leu plate supplemented with the optimal AbA concentration (700 ng/mL). In contrast, the co-transformants harboring the *pro2PHT1* or *pro3PHT1* baits did not grow under their respective selection pressures. The results showed that UpMYB-PHL IF can bind to the *UpPHT1* promoter by the P1BS motif.

To determine whether UpMYB-PHL can transactivate the expression of *UpPHT1*, a transient dual-luciferase reporter assay was conducted in *C. reinhardtii*. The *pro1PHT1* fragment was fused to the firefly luciferase (fLUC) reporter gene, while *UpMYB-PHL* driven by the 35S promoter served as the effector. Co-expression of the UpMYB-PHL effector and the *pro1PHT1*-LUC reporter resulted in a significant increase in the LUC/REN ratio compared with the negative control (empty effector + reporter) (*p* < 0.01) (Figure 5C). Taken together, these results indicated that UpMYB-PHL and the *UpPHT1* promoter could interact through the P1BS motif.

### 3.5. UpGAPDH and UpVPS53 Are Putative Interacting Proteins of UpMYB-PHL

**Figure 6:**
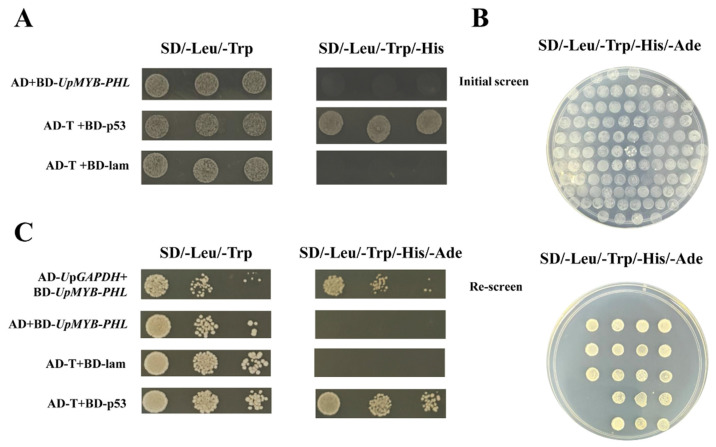
Screening and identification of UpMYB-PHL interacting proteins. (**A**) Assessment of the autoactivation and toxicity of UpMYB-PHL in the Y2H system. Yeast strains co-transformed with BD-*UpMYB-PHL* and AD vectors were spotted onto SD/-Leu/-Trp and SD/-Leu/-Trp/-His plates. Co-transformations of AD-T + BD-p53 and AD-T + BD-lam served as the positive and negative controls, respectively. (**B**) Screening and verification of UpMYB-PHL interacting proteins from a *U. prolifera* cDNA library by Y2H assay. Upper panel: initial screening of yeast colonies on SD/-Leu/-Trp/-His/-Ade plate. Lower panel: re-screening and validation of positive clones on the same selective plate. (**C**) Point-to-point verification of the interaction between UpMYB-PHL and UpGAPDH by Y2H assay. Co-transformant strains were adjusted to OD_600_ values of 0.2, 0.02 and 0.002 and then spotted onto SD/-Leu/-Trp and SD/-Leu/-Trp/-His/-Ade plates.

To investigate the autoactivation of UpMYB-PHL in the Y2H system, BD-*UpMYB-PHL* and AD were co-transformed into yeast AH109. The AD-T + BD-p53 strain served as the positive control, and the AD-T + BD-lam strain served as the negative control. The results showed that the experimental and negative control strains grew only on SD/-Leu/-Trp plate, indicating that UpMYB-PHL has no obvious autonomous activation (Figure 6A).

To elucidate the regulatory mechanism of UpMYB-PHL, BD-*UpMYB-PHL* was used as bait to screen for potential interacting proteins from a cDNA library of *U. prolifera*. As shown in Figure 6B, 96 single clones were randomly selected for primary screening, yielding 18 clones that grew on the SD/-Leu/-Trp/-His/-Ade plate. These 18 positive clones were sequenced and analyzed. Based on sequence alignment and functional annotation, two distinct candidate genes were identified. One sequence was annotated as vacuolar protein sorting-associated protein 53 (VPS53), whereas the other was identified as glyceraldehyde-3-phosphate dehydrogenase (GAPDH). These results suggest that UpGAPDH and UpVPS53 are candidate interacting proteins of UpMYB-PHL. These candidates were subsequently chosen for further validation.

### 3.6. UpMYB-PHL Physically Interacts with UpGAPDH

Based on the screening results described above, we cloned the full-length CDS of *UpGAPDH* to further validate its interaction with UpMYB-PHL. Subsequently, a Y2H assay was performed by co-transforming the bait construct BD-*UpMYB-PHL* and the prey construct AD-*UpGAPDH* into yeast strain AH109. All co-transformants grew normally on the SD/-Leu/-Trp plate, confirming successful transformation (Figure 6C). The negative control strains (BD-*UpMYB-PHL* + AD and BD-Lam + AD-T) did not grow on the SD/-Leu/-Trp/-His/-Ade plate. In contrast, the strains co-expressing UpMYB-PHL and UpGAPDH displayed robust growth and formed distinct colonies, with a growth phenotype identical to that of the positive control (BD-p53 + AD-T). These results indicate that UpMYB-PHL physically interacts with UpGAPDH.

## 4. Discussion

### 4.1. UpMYB-PHL Is a Member of MYB-CC TFs Family

The MYB TFs play significant roles in plant growth, development and stress responses [19,28]. At present, the function of MYB TFs has been extensively studied in higher plants and algae [20,21,29,30]. In general, MYB TFs are classified into four major groups according to the number of adjacent repeats in the MYB DNA-binding domain: 1R-MYB (MYB-related), 1R-MYB (R2R3-MYB), 3R-MYB (R1R2R3-MYB) and 4R-MYB [19]. And MYB proteins with a single MYB DNA-binding domain are classified as MYB-related families [31]. For example, PSR1 from *C. reinhardtii* and PHR1 of *A. thaliana* are characterized by the presence of a conserved coiled-coil (CC) domain, classified into an CC family [20,21]. In the study, UpMYB-PHL, an MYB TF, was isolated from *U. prolifera*, structural prediction reveal the UpMYB-PHL contains an intact MYB DNA-binding domain. And the MYB DNA-binding domain of UpMYB-PHL is highly homologous to the MYB-related proteins from green algae and higher plants. Hence, it is preliminarily determined that UpMYB-PHL belongs to the MYB-related family. Interestingly, the UpMYB-PHL contains a less conserved CC domain, just as has been observed in several other algae [32,33]. The CC domain of the MYB-related TF was initially believed to play a role in protein interactions, such as forming dimers or polymers, as well as mediate its interaction with other proteins [21,32]. Some later studies have shown that the CC domain may not be necessary, and the MYB domain alone is sufficient for target DNA recognition and binding [34]. In general, the CC domain mediates the interactions between proteins [35]. In this study, UpMYB-PHL harbors a low conserved CC domain, and similar lowly conserved CC domains are also present in other algae [32,33]. We speculate that the CC domain is less conserved compared to the DNA-binding domain.

Moreover, phylogenetic analysis revealed that UpMYB-PHL was clustered with MYB-CC family TFs from green algae and higher plants as a single branch and exhibiting the closest relationship with Psr1 of *C. reinhardtii*, confirming that UpMYB-PHL belongs to the MYB-CC TF family.

### 4.2. UpMYB-PHL Enhances the Salt Tolerance of Plants

MYB-related proteins have been reported as positive regulators in response to salt stress in plants [36,37]. For example, expression of the MYB-related gene *AhMYB30* from peanut (*Arachis hypogaea* L.) was induced by salt stress [38]. And in rice, an MYB-related gene (*OsRL3*) showed upregulated expression under salt-stress conditions [39]. Here, *UpMYB-PHL* gene’s expression was significantly induced by high-salt stress, indicating that the *UpMYB-PHL* gene also plays an important role in *U. prolifera*’s response to high-salt stress. To further verify the biological function of *UpMYB-PHL* in vivo, we heterologous overexpressed this gene in the model microalga *C. reinhardtii*. The results found that *UpMYB-PHL* gene’s expression level in transgenic strains was upregulated by salt stress. Moreover, its tolerance to salt stress was improved. This phenomenon is consistent with earlier reports that in an overexpressed MYB-related gene of *Lilium lancifolium* in *A. thaliana*, the salt stress resistance of transgenic plants was enhanced [40]. In another experiment, in an overexpressed MYB-related gene *AhMYB30* of peanut in *A. thaliana*, the salt stress tolerances of these plants was enhanced, in concert with the upregulation of stress-responsive gene expression [38]. Therefore, we propose that *UpMYB-PHL* may regulate downstream genes in the stress signaling pathways to enhance high-salt stress tolerance in *U. prolifera*.

### 4.3. The UpMYB-PHL-UpPHT1 Module Exhibits High Conservation During the Evolution of Photosynthetic Organisms

The MYB-CC family TF is one of the MYB-related subfamilies, as a phosphate (Pi) starvation response 1 (PHR1) protein is the central regulator of Pi starvation signals in plants, enabling plants to maintain Pi homeostasis [20,32]. The MYB-CC family TF exhibits significant conservation in both structural composition and key biological functions from unicellular microalgae (such as Psr1 in *C. reinhardtii*) to higher vascular plants (such as PHR1 in *A. thaliana*) [20,22]. Recently, research has revealed the MYB-CC also responds to abiotic stresses by regulating Pi homeostasis [41,42]. For example, in maize, ZmPHR1 regulates the expression of the phosphorus starvation responses gene to maintain Pi homeostasis, thereby improving the ability to combat drought [41]. And in rice, OsPHL7 is involved in maintaining Pi homeostasis, thereby enhancing the tolerance to Pi deficiency and salt stress [42]. In general, the MYB-CC family TF regulates directly or indirectly phosphate transporter (*PHT*) gene expression by binding to a PHR1-binding site (P1BS) for Pi uptake [20,43]. Therefore, we speculate that UpMYB-PHL may also regulate *PHT* homologous gene expression to performs its function. In this study, a *PHT* gene was identified from *U. prolifera*, namely *UpPHT1*, and sequence analysis indicated the *UpPHT1* promoter contains the sequence “GGCATATTC”, which is highly consistent with the classic P1BS core conserved sequence (GNATATNC) [22,44]. Moreover, we confirmed that UpMYB-PHL binds to the *UpPHT1* promoter by P1BS cis-acting element by Y1H and Dual-luc assays. These results indicate that UpMYB-PHL activates *UpPHT1* gene expression. Together with previous studies, we predict that the UpMYB-PHL-*UpPHT1* module enhances *U. prolifera*’s tolerance to high-salt stress by maintaining Pi homeostasis, and the PHL1-*PHT1* module exhibits high conservation during the evolution of photosynthetic organisms.

### 4.4. UpGAPDH Participates in High-Salt Stress Response in U. prolifera

**Figure 7:**
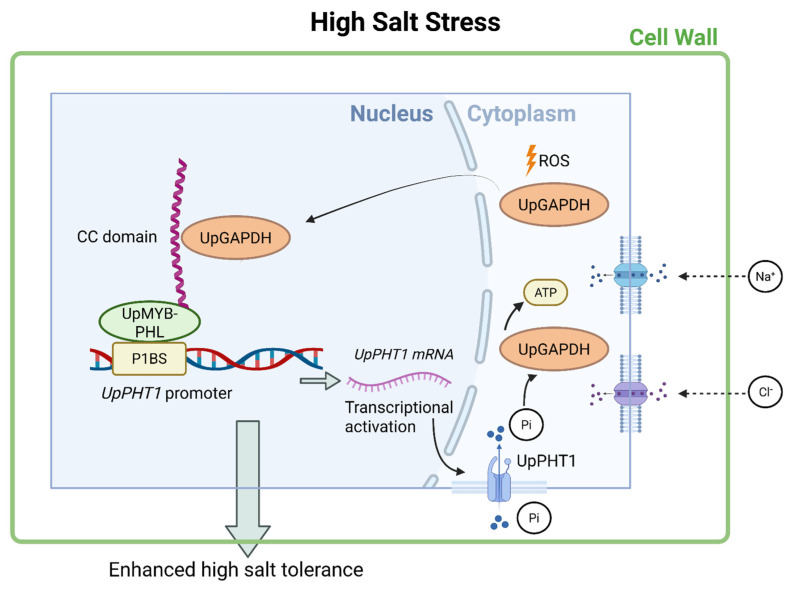
A working model of the UpMYB-PHL regulatory network under high-salt stress in *Ulva prolifera*. Under high-salt conditions, the core glycolytic enzyme UpGAPDH moves from the cytoplasm into the nucleus. Here, it physically interacts with the coiled-coil (CC) domain of the transcription factor UpMYB-PHL. This interaction helps UpMYB-PHL specifically bind to the P1BS element in the *UpPHT1* promoter, which quickly activates the transcription of the *UpPHT1* gene. By linking energy metabolism with phosphorus uptake, this regulatory mechanism significantly improves the salt tolerance of *U. prolifera*.

Glyceraldehyde-3-phosphate dehydrogenase (GAPDH), a classical glycolytic enzyme, is involved in cellular energy production [45]. However, studies have revealed that GAPDH is not only an enzyme but also a multifunctional protein playing crucial roles in abiotic stress responses in plants [46,47]. One of the moonlighting functions of GAPDH is its stress-promoted nuclear translocation [48,49]. In *A. thaliana*, GAPDH accumulated in the nucleus in response to cadmium and heat stress, and a nuclear factor Y subunit C10 (NFYC10) has been identified as a GAPDH-binding protein [48]. In rice, the oxidative stress induced nuclear accumulation of GAPDH, and the nuclear-localized GAPDH acts as a transcriptional activator, interacting directly with transcription factors and gene promoters, thereby modulating expression of the stress response gene [49]. In this study, we found GAPDH physically interacts with UpMYB-PHL in *U. prolifera*, and speculate that UpMYB-PHL might also be a target of GAPDH. In general, GAPDH plays a crucial role in plant defense, functioning not only as a key enzyme in glycolysis but also in regulating metabolic pathways to provide the substrates necessary for energy and the synthesis of defense-related compounds. Interestingly, while GAPDH possesses a nuclear export signal, it does not have a nuclear localization signal (NLS), suggesting that other mechanisms are involved in its translocation to the nucleus [50]. And translocation from the cytosol to non-cytosolic compartments leads to the acquisition of distinct functions beyond its traditional glycolytic role [47]. In potato, Snakin-2, an antimicrobial peptide, interacts with GAPDH and enhances GAPDH activity, impacting energy metabolism and delaying tuber germination [51,52]. Therefore, we hypothesize that UpGAPDH cooperates with UpMYB-PHL to regulate the expression of downstream genes in nuclei, thereby enhancing *U. prolifera*’s tolerance to salt stress. This study reveals a novel salt-tolerance mechanism in U. prolifera coordinated by the transcription factor UpMYB-PHL (Figure 7). By simultaneously activating the phosphate transporter UpPHT1 and interacting with the metabolic enzyme UpGAPDH, UpMYB-PHL effectively links phosphorus uptake with rapid energy production. This dual-action strategy provides the essential materials and ATP for the algae to grow and develop under severe salt stress.

Based on these results, we propose a regulatory model: high-salt stress promotes translocation of a portion of the cytoplasmic UpGAPDH into *U. prolifera* nuclei, where UpGAPDH is associated with the transcription factor UpMYB-PHL to increase the expression of the *PHT1* gene. Ultimately, the cells absorb large quantities of Pi, leading to the production of abundant ATP via UpGAPDH. The surplus Na^+^ and C^−^ are then expelled to the outside to preserve ionic homeostasis, thereby increasing *U. prolifera*’s tolerance to high-salt stress.

## 5. Conclusions

The current study reveals a novel salt-tolerance mechanism in *U. prolifera* coordinated by the transcription factor UpMYB-PHL (Figure 7). Expression analysis and heterologous overexpression of UpMYB-PHL in the model microalga prove it is involved in the salt stress response of algae. Moreover, UpMYB-PHL’s coordination of the phosphorus transporter and energy metabolism in *U. prolifera*’s response to salt stress was revealed. The results of this study provide a new perspective for understanding the salt stress resistance of *U. prolifera*.

## Data Availability

The original contributions presented in this study are included in the article/Supplementary Materials. Further inquiries can be directed to the corresponding author.

## Supplementary Materials

The following supporting information can be downloaded at: https://www.mdpi.com/article/10.3390/biology15131050/s1, Table S1: Primers and sequences used in this experiment; Table S2: The protein sequence for Figure 1D; File S1: Full-length, uncropped Western blots for Figure 3C.
