# Supplementary material for: An MYB-Related Transcription Factor, UpMYB-PHL, Is Involved in Salt Tolerance by Coordinating Phosphorus Transporter and Energy Metabolism in Ulva prolifera
Source: Biology (Basel). 2026 Jul 1;15(13):1050. doi: 10.3390/biology15131050 (PMC13360470; doi:10.3390/biology15131050)
Supplement: Supplementary file 1 [file biology-15-01050-s001.zip › biology-4396087-File S1.pdf]

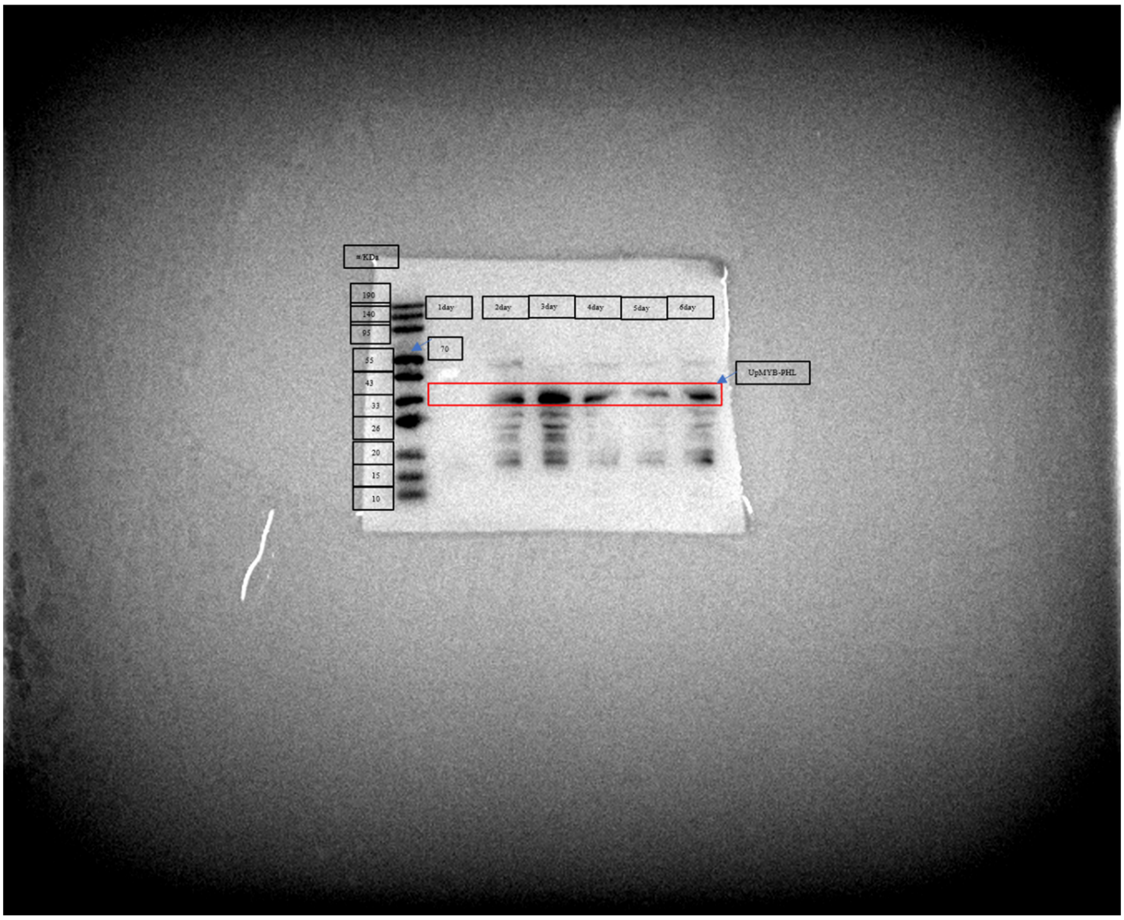

Figure S1. Western blot membrane of UpMYB-PHL-His (~33 kDa) protein detected with anti-His (AB3071; 1:1000; Beyotime Biotechnology, Shanghai, Chian) antibody. Gel-separated proteins were transferred to nitrocellulose membranes (0.2 µm pore size; Beyotime) by semi-dry electroblotting (1.5 mA per cm^2, 20 min). Membranes, incubated with a horseradish peroxidase (HRP)-conjugated secondary antibody (A0216; 1:1000; Beyotime), were developed with BeyWB™ 30 min Western Assay Kit (Beyotime). #Weight marker (molecular weight in kDa): Blue Plus V Protein Marker (DM141-01, Transgene), 10 to 190 kDa. UpMYB-PHL protein levels of *UpMYB-PHL* OE stains under high-salt stress at different indicated time (1st day, 2nd day, 3rd day, 4th day, 5th day, and 6th day) were detected by WB.

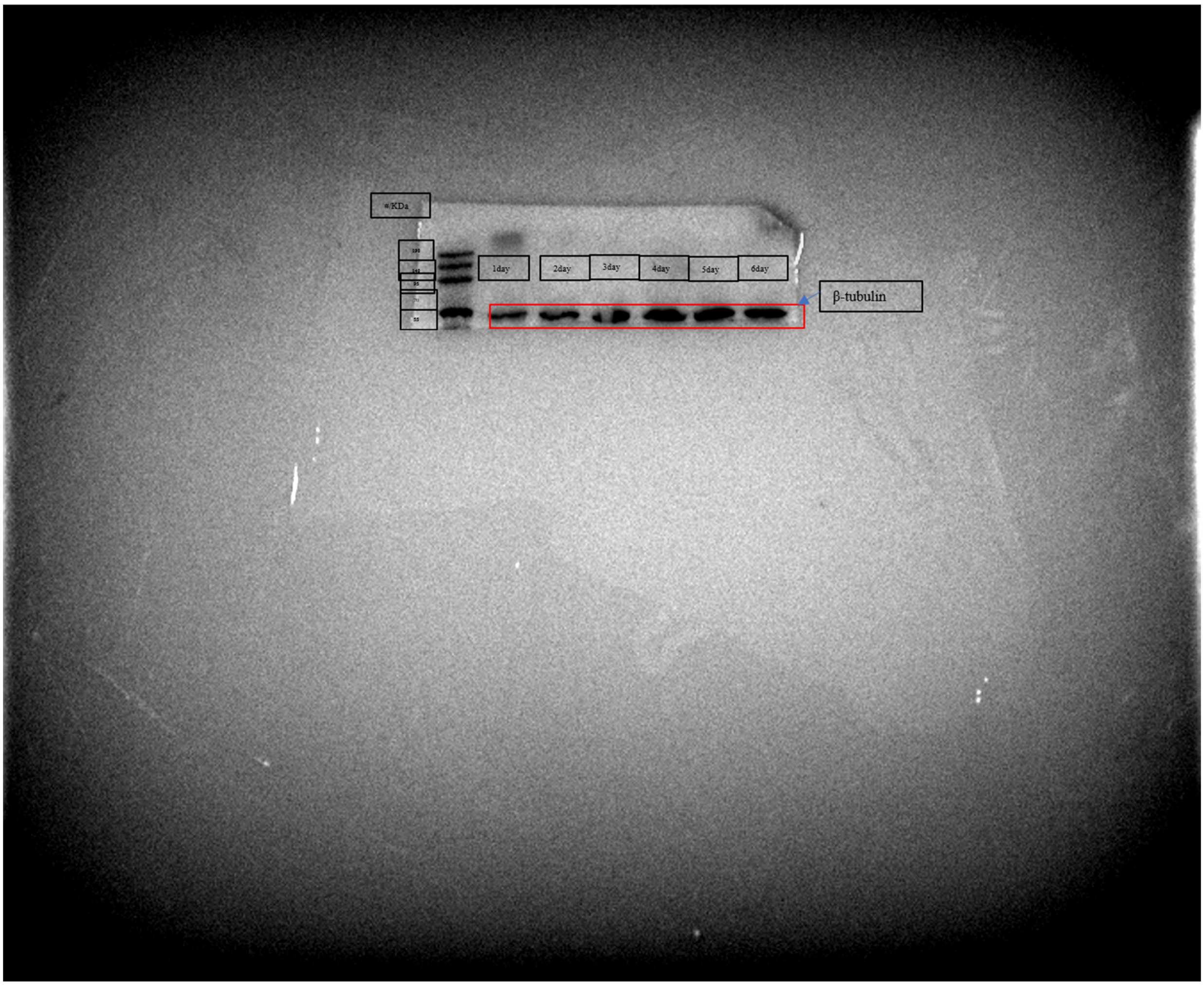

Figure S2. Western blot membrane of β-tubulin (~55 kDa) protein detected with anti-β-tubulin (AF2835; 1:1000; Beyotime Biotechnology, Shanghai, Chian) antibody. Gel-separated proteins were transferred to nitrocellulose membranes (0.2 μm pore size; Beyotime) by semi-dry electroblotting (1.5 mA per cm^2, 20 min). Membranes, incubated with a horseradish peroxidase (HRP)-conjugated secondary antibody (A0216; 1:1000; Beyotime), were developed with BeyWB™ 30 min Western Assay Kit (Beyotime). #Weight marker (molecular weight in kDa): Blue Plus V Protein Marker (DM141-01, Transgene), 10 to 190 kDa. β-tubulin protein of *UpMYB-PHL* OE stains under high-salt stress at different indicated time (1st day, 2nd day, 3rd day, 4th day, 5th day, and 6th day) were detected by WB.

Notes: Since the size of β-tubulin protein is approximately 55 kDa, the membrane of the Figure S1 was washed with the washing solution to remove the antibodies. And the Figure S2 was obtained from the upper part of Figure S1, which was incubated with the β-tubulin antibody.
